# Cell wall associated immunity in plants

**DOI:** 10.1007/s44154-021-00003-4

**Published:** 2021-08-18

**Authors:** Jiangxue Wan, Min He, Qingqing Hou, Lijuan Zou, Yihua Yang, Yan Wei, Xuewei Chen

**Affiliations:** 1 State Key Laboratory of Crop Gene Exploration and Utilization in Southwest China, Sichuan Agricultural University at Wenjiang, Chengdu, 611130 Sichuan China; 2 Ecological Security and Protection Key Laboratory of Sichuan Province, Mianyang Normal University, Mianyang, 621000 Sichuan China

**Keywords:** Cell wall, Oligosaccharides, Elicitor, Plant immunity, Disease resistance

## Abstract

The plant cell wall is the first physical and defensive barrier against pathogens. The plant cell wall usually undergoes dynamic remodeling as an immune response to prevent infection by pathogens. In this review, we summarize advances on relationship between cell wall and immunity in plants. In particular, we outline current progresses regarding the regulation of the cell wall components, including cellulose, hemicellulose, pectin and lignin, on plant disease resistance. We also discuss the impacts of cell wall-derived cellodextrin, oligogalacturonic acid and xyloglucan/xylan oligosaccharides as potent elicitors or signal molecules to trigger plant immune response. We further propose future studies on dissecting the molecular regulation of cell wall on plant immunity, which have potentials in practical application of crop breeding aiming at improvement of plant disease resistance.

**Supplementary Information:**

The online version contains supplementary material available at 10.1007/s44154-021-00003-4.

## Introduction

Plants are consistently confronted with biotic stresses imposed by insects or pathogens during their growth within the natural environments (Panstruga et al. 2009). These stressful restrictions force plants to develop cell-autonomous monitoring systems to cope with the harsh conditions for balancing the stress response and their growth (Hofte and Voxeur 2017; Engelsdorf and Hamann 2014). Plants have therefore evolved complicated and effective mechanisms with the long-term adaptation to resist stresses (Glazebrook 2005). The plant cell wall acts as the first physical barrier to defend against invasion of pathogens, and it is also involved in sensing external stresses and transferring the corresponding signal to stimulate defense responses (Cosgrove 2005). The cell wall is often regarded as a passive barrier upon pathogens attack, but recently, growing evidence has shown that it more likely functions initiatively.

The structure and component of cell wall vary with plant species, developmental stage and response to stress (Scheller and Ulvskov 2010). As an important monitoring system, the plant cell wall undergoes dynamic remodeling in adaption to external stresses including pathogenic infection by microbes (Zhao and Dixon 2014). Reinforcement of cell wall has been confirmed as a typical physiological consequence of activated immune response in plants (Luna et al. 2010; Underwood 2012). Emerging evidence indicates that the cell wall integrity (CWI) maintenance is important for activating and monitoring the defense responses (Vaahtera et al. 2019). In this regard, alternations of the plant cell wall are considered to interconnect with the two-layered innate immune system composed of the pathogen-associated molecular pattern-triggered immunity (PTI) and effector-triggered immunity (ETI). PTI is the first layer of immunity which is activated when the host pattern recognition receptor (PRR) senses the pathogen-associated molecular pattern (PAMP). To evade PTI, microbial pathogens secrete effectors to dampen plant defense response. Plants have accordingly evolved resistance proteins, typically those with nucleotide binding site-leucine-rich repeat (NLR) domains, to recognize pathogens’ effectors to initiate the second layer of defense called ETI (Jones and Dangl 2006). Among increasing knowledge on regulatory mechanism of PTI and ETI, emerging evidence shows that the component and modification of cell wall are associated with plant immunity (Nicaise et al. 2009; Wolf et al. 2012). Here, we review current progresses concerning the modulation of cell wall on plant immunity.

## Cell wall synthesis and structure

**Fig. 1:**
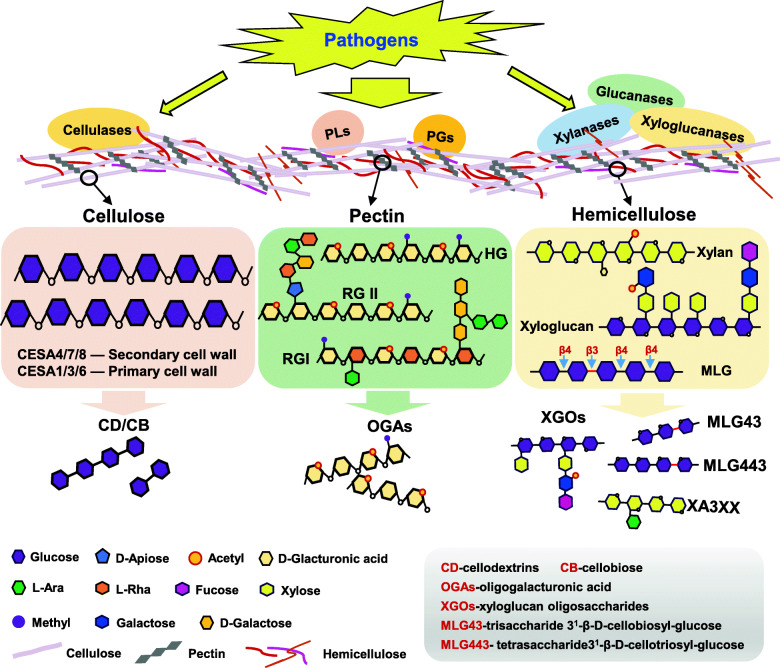
Structure of cell wall polysaccharides and their degradation by cell-wall degrading enzymes (CWDEs). Polysaccharides make up most of the cell wall and vary in different species and tissues. Cellulose is the core load-bearing component of cell wall and composed of β-1,4-linked glucan. Pectin provides a matrix that embeds the cellulose-hemicellulose network. The four types of pectin polysaccharides including homogalacturonans, rhamnogalacturonan-I, rhamnogalacturonan-II and xylogalacturonan are abbreviated as HG, RG-I, RG-II and XGA, respectively. Xylan and mixed-linked glucans are the main components of monocots hemicellulose while the xyloglucan is the major hemicellulose in dicots. To overcome the barrier of the plant cell wall, pathogens produce CWDEs to degrade plant cell wall polysaccharides, resulting in the release of cell wall oligosacchrides like cellodextrins and cellobiose from cellulose, oligogalacturonic acids from pectin, the xyloglucan oligosaccharides, 3^3^-α-L-arabinofuranosyl-xylotetraose (XA3XX) from arabinoinxylan and the D-cellobiosyl-(1,3)-β-D-glucose (MLG43) from mixed-linked glucans

The plant cell wall is a complex network composed of various polysaccharides. The presence of cellulose, hemicellulose and pectin is a common feature for all plants’ cell wall (Cosgrove 2005), which is generally divided into two types. Cellulose microfibril is similarly present in type I and type II cell wall. The main difference in component between the two types of cell wall is the non-cellulosic polysaccharides. Xyloglucans is the main non-cellulosic polysaccharide in type I cell wall, while arabinoxylan and β-(1➝3, 1➝4) mixed-linkage glucan (MLG) are predominant in type II cell wall (Wolf et al. 2012). The type I cell wall is mainly present in dicots including *Arabidopsis thaliana*, while the type II exits in monocots like poaceae (Carpita and Gibeaut 1993). Cellulose accounts for about one-third of the cell wall biomass (Fig. 1), thus constituting a key component of the plant cell wall and being regarded as the most abundant biological polymer on earth (Zhong et al. 2019). The cellulose filament is formed by 36 unbranched β-1,4-glucan chains through hydrogen bonding and intermolecular forces (Taylor 2008). The compacted cellulose filaments serve as the basic skeleton of cell wall to allow for mechanical properties in plant (Somerville 2006).

Hemicellulose is another major component of plant cell wall (Fig. 1). It is made up of diverse polysaccharides including xylan, xyloglucan, mannan, glucomannan and MLG. Hemicellulose polysaccharides confer strength to the cell wall by interacting with cellulose and lignin (Scheller and Ulvskov 2010). Arabinoxylan and MLG make up most of the hemicellulose in poaceae (Carpita and Gibeaut 1993). Among the different polysaccharides in dicots hemicellulose, xyloglucan is the main component in primary cell wall. The basic structure of xyloglucan is composed of β-(1➝4)-glucan backbone which contains xylose as residues at *O*-6 position of glucose. The xylose within xyloglucan can further be decorated with galatosyl and galatosyl-fucosyl residues. Those decorations usually vary between plant species (Park and Cosgrove 2015; Schultink et al. 2014).

Pectin is formed by various polysaccharides and its main chain is α-1,4-galacturonic acid (Fig. 1). The content of pectin varies between different plants. For example, pectin accounts for approximately 35% of primary cell wall in dicots and non-graminaceous monocots, whereas accounts for only 2-10% of gramineae such as rice (O'Neill and Albersheim 1990; Ridley et al. 2001). There are mainly four different types of pectin polysaccharides, viz homogalacturonan (HG), rhamnogalacturonan-I (RG-I), rhamnogalacturonan-II (RG-II) and xylogalacturonan (XGA). Homogalacturonan, as a homopolymer of α-1, 4-galacturonic acid, is the most abundant pectin by occupying about 60% (Ridley et al. 2001; Caffall and Mohnen 2009). Compared to the homogeneity of homogalacturonan, RG-II is composed of α-1,4-galacturonic acid containing complicated substitutions, while RG-I is the only pectin polysaccharides with mixed backbone of disaccharide (α-1,4-D-GlaA-α-1,2-L-Rha) and with substitutions on rhamnose (Ridley et al. 2001; Harholt et al. 2010). RG-II and RG-I occupies approximately 10% and 20-35% of pectin respectively (Arana et al. 2009). Xylogalacturonan has xylose substituents at the *O*-3 position of the main chain of galacturonic acid, and it constitutes minor proportion of pectin (Ridley et al. 2001).

Compared to the cellulose-based plant cell wall, fungal cell wall mainly consists of an outer layer of glycoproteins and an inner skeletal layer of gluca and chitin (Arana et al. 2009). Proteins occupy about 35-40% of total fungal cell wall in content (Fontaine et al. 2000). Most of the fungal cell wall proteins are glycoproteins modified with *N*- and *O*-linked oligosaccharides. The oligosaccharides form different structures in fungi, but their main components are mannan and galactomannan (Jin 2012). As the major structural polysaccharides of fungal cell wall, glucan accounts for about 50-60% of the cell wall by dry weight (Free 2013). The β-1,3-glucan, mixed β-1,3-/1,4-glucan, β-1,6- and α-1,3-glucan are present in fungal cell wall. The major cell wall glucan is β-1,3-glucan which constitutes about 65-90% in content (Fontaine et al. 2000; Klis et al. 2001; Aimanianda et al. 2009; Grun et al. 2005). Chitin is a homopolymer of β-1,4-*N*-acetylglucosamine, making up approximately 1%-15% of the cell wall content (Free 2013).

In bacteria, peptidoglycan is the central component of cell wall. The peptidoglycan not only maintains the shape of bacterial cell, but also confers strength to counteract the osmotic turgor (Smith 2006; Vollmer et al. 2008). The bacterial peptidoglycan is composed of linear glycan crosslinked with short polypeptides (Fisher and Mobashery 2020). Specifically, the glycan strands are composed of alternating *N*-acetylglucosamine and *N*-acetylmuramic acid residues. The D-lactoyl group of *N*-acetylmuramic acid residue is further substituted by a peptide stem (Vollmer et al. 2008). In the gram-negative bacteria, a thin peptidoglycan layer is enclosed by two lipid bilayer membranes. In contrast, the gram-positive bacteria only contain a single membrane surrounded by a more cross-linked and thicker peptidoglycan layer (Shaku et al. 2020).

## Regulation of different cell wall components on plant immunity

Alterations in the composition, or structure of the cell wall have been demonstrated to affect plant resistance against biotic stresses. Here we summarize the plant disease phenotypes caused by alteration of cell wall polysaccharides and the underlying mechanisms (Additional file 1: Table 1).

### CWI maintenance and plant immunity

CWI impairment is usually caused by damage in the physical structure of cell wall during development or exposure to diverse stresses. Upon the invasion of pathogens, CWI system perceives the changes of cell wall status to activate defense responses (Gigli-Bisceglia et al. 2020). Several families of plant protein are involved in detecting cell wall damage (Bacete and Hamann 2020). The leucine-rich repeat receptor kinase MIK2 is an important regulator of CWI triggered by cellulose biosynthesis inhibition (Van der Does et al. 2017). MIK2 affects disease resistance, as the mutant *mik2-1* exhibits decreased resistance to the fungal pathogen *Fusarium oxysporum*. (Van der Does et al. 2017). THE1, a member of *Catharanthus roseus* Receptor Like Kinase 1-like (CrRLK1L) protein family, is originally identified in response to the CWI impairment caused by the reduction of cellulose (Hematy et al. 2007). THE1 functions upstream of GEF4 (Guanosine nucleotide exchange factors 4) to positively regulate defenses responses of Arabidopsis against *Botrytis cinerea* infection (Qu et al. 2017).

As another member of the CrRLK1L family, FER positively regulates the PAMP-triggered immunity through the CWI pathway. The extracellular domain of FER is able to bind to pectin-derived cell wall fragments *in vitro* (Lin et al. 2018). FER also acts as the receptor for rapid alkalinization factors peptides related to the alkalinization and growth inhibition (Haruta et al. 2014; Stegmann et al. 2017). The *fer-4* mutant displays enhanced resistance against *F. oxysporum*, indicating the negative regulation of FER on defending fungal infection *(*Masachis et al. 2016*)*. However, FER positively regulates defense against *Pseudomonas syringae* by promoting the formation between FLS2 (Flagellin sensitive2)/EFP (Elongation factor tu receptor) and their co-receptor BAK1 (Brassinosteroid insensitive 1-associated kinase1) (Stegmann et al. 2017). ANX1 and ANX2 are two extra members of CrRLK1L family. Theynegatively regulate PTI and NLR-mediated immunity. Specifically, *anx1*, *anx2* and *anx1 anx2* mutants show increased resistance to *P. syringae* due to ROS accumulation and MAPK activation (Mang et al. 2017).

### Impact of cellulose on plant immunity

In higher plants, cellulose is synthesized by a cellulose synthase complex, which is usually located on plasma membrane as heterohexamer in the form of rosette architecture (Desprez et al. 2007). Each unit of hexamer composes of six catalytic submits abbreviated as CESAs, and requires at least three different CESAs to work synergistically to produce cellulose (Desprez et al. 2007). A full cellulose synthase complex therefore contains 36 cellulose synthase. CESA1, CESA3 and CESA6 are responsible for synthetizing cellulose in the primary cell wall, while CESA4, CESA7 and CESA8 are involved in the production of secondary cell wall cellulose (Somerville 2006; Robert et al. 2004; Persson et al. 2007; Taylor et al. 2004).

As the central component of cell wall, cellulose affects both growth and defense response of plants. Plant mutants impaired in cellulose synthesis exhibit severe developmental defects such as dwarfing and reduced yield. In most cases, those mutants are increased in disease resistance, indicating the trade-offs between growth and disease resistance (Schulze et al. 2010; Ramirez et al. 2011). For example, the Arabidopsis mutant *irx5/3/1* (*irregular xylem*) correspondingly disrupted with the *CESA4/7/8*, displays structural changes in the secondary cell wall and enhanced disease resistance against the bacterial pathogen *Ralstonia solanacearum* and necrotrophic fungal pathogen *Plectosphaerella cucumerina* (Hernandez-Blanco et al. 2007). The expression of *CESA4/7/8* is positively regulated by a transcription factor *MYB46*. In consistence with the role of *CESA4/7/8* on plant disease resistance, loss-of-function of *MYB46* increases disease resistance of Arabidopsis to *B. cinerea (*Ramirez et al. 2011*)*. In few cases, however, blockage in cellulose synthesis decreases plant disease resistance. For instance, when silenced for *HvCSLD2* encoding cellulose-synthase-like D, barley displays reduced cellulose content in epidermal cell wall and its papillae is more easily penetrated by fungus *Blumeria graminis*, thereby leading to compromised resistance to the powdery mildew (Douchkov et al. 2016).

The impairment of cellulose can activate defense responses such as hormones and callose deposition. CESA3-deficient mutant of Arabidopsis shows increased resistance against *P. syringae*, *B. cinerea* and *Erysiphe cichoracearum* and it is activated in both ethylene (ET) and jasmonic acid (JA) signaling pathways (Hernandez-Blanco et al. 2007; Ellis et al. 2002). In tomato, the endo-β-1,4-glucanase family proteins Cel1 and Cel2 negatively regulates resistance against *B. cinerea*. Their regulation is associated to SA- and JA-dependent defenses (Flors et al. 2007). Another endo-β-1,4 glucanase, encoded by *KORRIGAN1* in Arabidopsis, is involved in disease response to pathogens, as shown by the fact that the mutant *kor1-1* lacking *KORRIGAN1* is more susceptible to infection by *P. syringae* and accumulates more JA than wild type plant upon infection (Lopez-Cruz et al. 2014). Chemical inhibition of cellulose synthesis also leads to the production of JA, ROS and the deposition of lignin to affect plant immunity (Ellis et al. 2002; Mélida et al. 2015; Hamann et al. 2009).

### The hemicellulose-mediated plant immunity

Xylan and xyloglucan both belong to hemicellulose and are also main components of the plant cell wall. Growing evidence demonstrates that changes in xylan or xyloglucan affect disease resistance of Arabidopsis to pathogens (Delgado-Cerezo et al. 2012; Sampedro et al. 2010; Chowdhury et al. 2017). The Arabidopsis mutants *det3* (*de-etiolated3*) and *irx6-1* (*irregular xylem 6-1*) mutants contain more xylose in the cell wall than that of the wild type. These mutants are both enhanced in resistance to the necrotic fungu*s P. cucumerina* (Delgado-Cerezo et al. 2012; Rogers et al. 2005; Brown et al. 2005). The Arabidopsis *xyl1-2* mutant lacking an α-xylosidase displays modified xyloglucan modification and is increased in resistance to *P. cucumerina (*Delgado-Cerezo et al. 2012*;* Sampedro et al. 2010*)*. Similarly, overexpression of xylan synthesis-related glycosyltransferase in barley improves the resistance against powdery mildew (Chowdhury et al. 2017). Moreover, the heterotrimeric G-protein subunits G*β* (encoded by *AGB1*) and G*γ* (encoded by *AGG1/AGG2*) are both crucial for maintaining xylose content in the cell wall of Arabidopsis; their corresponding deletion mutants *agb1-2* and *agg1 agg2* are reduced in resistance to several pathogens (Delgado-Cerezo et al. 2012; Brenya et al. 2016; Trusov et al. 2006).

Hemicellulose polysaccharides, such as xylan and xyloglucan, often undergo acetylation to regulate plant development (Pauly et al. 2001). At least two protein families, viz the reduced wall acetylation (RWA) and trichiome birefringence-like (TBL) families, are involved in the acetylation of hemicellulose (Manabe et al. 2011; Gille et al. 2011). RWA family proteins function as *O*-acetyltransferase at the initial stage of acetylation on hemicellulose and other polysaccharides (Manabe et al. 2013). The TBL family proteins tend to acetylate specific polysaccharides compared to RWA proteins (Gille et al. 2011; Xiong et al. 2013; Yuan et al. 2013; Gao et al. 2017; Stranne et al. 2018). However, how RWA and TBL proteins function together is still unclear, it seems more likely that RWA proteins produce acetylated intermediate products which act as substrates of TBL proteins (Persson et al. 2007).

Hemicellulose acetylation affects biotic invasion by determining the cross-link of polysaccharides in the cell wall (Gille and Pauly 2012). In Arabidopsis, the *rwa2* mutant is deficient in a potential *O*-acetyltransferase and displays increased resistance to *B. cinerea (*Manabe et al. 2011*)*. The mutant *pmr5* (*powdery mildew resistant 5*) is down-regulated in *TBL44* gene expression, leading to significant increase in resistance to powdery mildew but unaltered resistance to *Pseudomonas. syringae* (Gille et al. 2011; Vogel et al. 2004; Engelsdorf et al. 2017). A TBL member gene *ESK1* encodes an *O*-acetyltransferase involved in xylan acetylation in Arabidopsis. The *esk1* mutant shows reduction in xylan acetylation and is increased in resistance against *P. cucumerina (*Urbanowicz et al. 2014*;* Escudero et al. 2017*)*. With respect to rice, *OsTBL1* and *OsTBL2* are co-expressed with many *PR* genes, and their double deletion mutant *tbl1 tbl2* is reduced in resistance to leaf blight disease (Gao et al. 2017). A recent study showed that in face of insect-induced damage, JA signaling is activated in Arabidopsis and expression of *AtTBL37* is elevated, consequently increasing acetylated polysaccharides to thicken secondary cell wall and enhance resistance against herbivore (Sun et al. 2020).

Xylan acetyl esterase (AnAXE) derived from the fungus *Aspergillus nidulans* also contributes to plant disease resistance, because overexpression of *AnAXE* in either Arabidopsis or *Brachypodium distachyon* reduces the degree of cell wall acetylation but increases plant resistance to fungal pathogens *B. cinerea* and *Bipolaris sorokiniana (*Pogorelko et al. 2013*)*. However, transgenic Arabidopsis and *B. distachyon* overexpressing *AnAXE* has no effect on resistance against neither the bacterial pathogen *P. syringae* nor *Xanthomonas oryzae* (Pogorelko et al. 2013). These also suggest that expression of *AnAXE* in dicotyledon and monocotyledon triggers different disease resistance responses to deal with fungal and bacterial pathogens.

### Effect of pectin on plant immunity

After pathogens break the outmost cutin layer of plant epicuticle, pectin serves as the first barrier to prevent invasion. Owing to this fact, alterations in pectin component or modification affect plant disease response. UDP-D-glucuronate 4-epimerase (GAE) controls pectin content by catalyzing epimerization of UDP-α-D-glucuronic acid to produce the monomeric precursor of pectin UDP-α-D-galacturonic acid (Gu and Bar-Peled 2004). In Arabidopsis, the *gae1 gae6* double mutant is impaired in producing pectin homogalacturonan and rhamnogalacturonan-I (Bethke et al. 2016). This mutant displays defects in resistance to pathogens including *P. syringae* and *B. cinerea*, and is hyper-responsive to the hormone jasmonic acid, indicating a link between pectin-mediated disease resistance and JA signaling. Pectin content of Arabidopsis is increased in the mutants *pmr5* and *pmr6*, conferring the mutants with enhanced disease resistance by reducing penetration of powdery mildew fungus (Vogel et al. 2004; Vogel et al. 2002). Similarly, the Arabidopsis mutant *mur8-1*, which is deficient in 4-GalA linkage, shows reduction in content of pectin rhamnogalacturonan-I and is therefore hyper-susceptible to the hemibiotrophic fungus *Colletotrichum higginsianum* (Engelsdorf et al. 2017; Mertz et al. 2012). In Arabidopsis, a polygalacturonase encoding gene *ADPG2* catalyzes depolymerization of homogalacturonan to negatively regulates disease resistance against the bacterial pathogen *P. syringae (*Wang et al. 2017*)*.

Pectin is generally secreted from the Golgi apparatus to the cell wall in a highly methylated form. Pectin methylesterases (PMEs) remove the methyl group of homogalacturonan during incorporation of pectin into the cell wall (Harholt et al. 2010; Sterling et al. 2001). It has been suggested that highly-methylated pectin shows well tolerance to the attack by cell-wall degrading enzymes (CWDEs) secreted by pathogens, thus conferring plant with disease resistance (Raiola et al. 2010). For instance, *PME3* disruption in Arabidopsis increases pectin methylesterification and enhances resistance to *B. cinerea* and *Pectobacterium carotovorum* due to less colonization of the two pathogens (McMillan et al. 1993); the degree of methylesterification in potato cultivars is positively correlated with their resistance to *P. carotovorum* (Lionetti et al. 2012). Because plants with increased methylesterification of pectin often possess enhanced resistance against pectinase released by pathogens, increasing pectin methylesterification is an ideal breeding strategy to improve crop disease resistance.

The expression of pectin methylesterase is subjected to post-transcriptional regulation by pectin methylesterase inhibitor (PMEI) (Wolf et al. 2009). Overexpression of PMEI has been shown to enhance plant resistance against various pathogens including fungi, bacteria and viruses (Lionetti et al. 2007; Lionetti et al. 2014). When overexpressing PMEI1 and PMEI2, Arabidopsis displays resistance to both powdery mildew and soft rot disease owing to increased pectin methylesterification (Lionetti et al. 2007). Similarly, heterogenous overexpression of kiwi PMEI enhances wheat resistance against *B. sorokiniana* and *Fusarium graminearum* (Volpi et al. 2011). In contrast, Arabidopsis overexpressing PMEI are extremely sensitive to virus infection (Lionetti et al. 2014). These studies demonstrate that modification of pectin such as methylesterification can modulate plant disease resistance and suggest that different degrees of pectin methylesterification have different effects on disease resistance.

PMEI can also act against microbial enzymes. CaPMEI1 from pepper (*Capsicum annuum*) exhibits antifungal activity *in vitro* against *F. oxysporum* f. sp. *matthiole*, *Alternaria brassicicola,* and *B. cinerea (*An et al. 2008*)*. Virus-induced silencing of CaPMEI1 confers pepper with decreased resistance to *X. campestris* pv. *vesicatoria* (An et al. 2008). In cotton (*Gossypium hirsutum*), GhPMEI3 efficiently inhibits the activity of GhPME2/GhPME31 and is able to increase cotton resistance to *Verticillium dahlia* via repressing the expression of a fungal polygalacturonase encoding gene *VdPG1* (Liu et al. 2018). In Arabidopsis, PMEI13 is regarded as a resistance factor against aphid infestation by influencing the degree of plant pectin methylesterification during aphid settling and feeding (Silva-Sanzana et al. 2019).

Pectin harbors another important modification called acetylation which occurs during pectin exocytosis during its incorporation into the cell wall (Scheller and Ulvskov 2010; Pauly and Scheller 2000). Two types of enzymes modulate acetylation degree of pectin. Pectin acetyltransferases is the first one transferring acetyl residues to polysacchrides, while pectin acetylesterase (PAE) is the other one cleaving acetyl groups from pectin such as galacturonic acid (Manabe et al. 2011; Lee et al. 2011; Bordenave et al. 1995). There is paucity of evidence showing the involvement of pectin acetylation in response to plant biotic stresses. *PAE4* and *PAE2* may play a role in plant defense (Philippe et al. 2017). *PAE4* expression is upregulated in Arabidopsis challenged with *B. cinerea, Hyaloperonospora arabidopsis* and *Phytophthora infestans. PAE2* is similarly induced in expression upon biotic stress. A recent study showed that pectin acetylesterases CsPAE2 negatively regulates *Citrus sinensis* resistance against bacterial canker disease, because knocking-down *CsPAE2* allows the disease agent *Xanthomonas, citri* to proliferate rapidly in citrus (Li et al. 2020a).

### Lignin-associated disease resistance

Lignin, a hydrophobic aromatic polymer, is usually present on the secondary cell wall of vascular plants (Chen and Dixon 2007). The phenylala-nine ammonia-lyase PAL1 acts as the first enzyme to produce precursors for lignin biosynthesis (Miedes et al. 2014). There are four members of *PAL* genes in Arabidopsis. Among these members, *PAL1* and *PAL2* have redundant function. Both the *pal1 pal2* double mutant and *pal1 pal2 pal3 pal4* quadruple mutant exhibit significant reduction in lignin accumulation and decreased resistance to *P. syringae (*Raes et al. 2003*;* Huang et al. 2010*;* Rohde et al. 2004*)*. In rice, *OsPAL1* mRNA is accumulated in the *bsr-k1* mutant which is disrupted of a tetratricopeptide repeats (TPRs)-containing protein required for modulating turnover of *OsPAL1* mRNA (Zhou et al. 2018). The elevated amounts of *OsPAL1* mRNA in the *bsr-k1* mutant leads to enhanced lignin content and confers broad-spectrum resistance to blast disease and bacterial leaf blight, which is caused by fungal pathogen *Magnaporthe oryzae* and *X. oryzae* respectively *(*Zhou et al. 2018*)*.

Lignin content is typically positively correlated with plant disease resistance. Higher lignification of the cell wall has been observed in plants exposed to pathogen infection or deficient in cellulose biosynthesis, thereby increasing mechanical strength of the plant cell wall and improving tolerance of the cell wall towards CWDEs released by pathogens (Hernandez-Blanco et al. 2007; Huckelhoven 2007). The rice transcription factor *OsMYB30* is able to activate the expression of lignin biosynthesis-associated genes *Os4CL3* and *Os4CL5*, resulting in accumulation of lignin subunits G and S to strengthen the sclerenchyma cell and resist *M. oryzae* penetration (Li et al. 2020b). However, there is also a scenario in which lignin content is negatively related to disease resistance. Down-regulation of lignin in the *Medicago sativa L*. by disrupting the gene encoding shikimate hydroxycinnamoyl transferase (HCT) increases alfalfa resistance against *Colletotrichum trifolli*, owing to activation in the defense response as manifested by the increase of pectin fragments release, up-regulation of *PR* genes and elevation of phytohormone levels (Gallego-Giraldo et al. 2011). In cotton, MYB4 functions as a negative regulator of lignin biosynthesis. When heterogeously expressed in Arabidopsis, MYB4 reduces lignin production and promotes release of oligogalacturonides, thereby activating JA biosynthesis and defense responses to confer enhanced disease resistance against the soil-borne fungus *V. dahliae* (Xiao et al. 2021).

## Cell wall-derived oligosaccharides as signaling molecules to trigger plant immunity

**Fig. 2:**
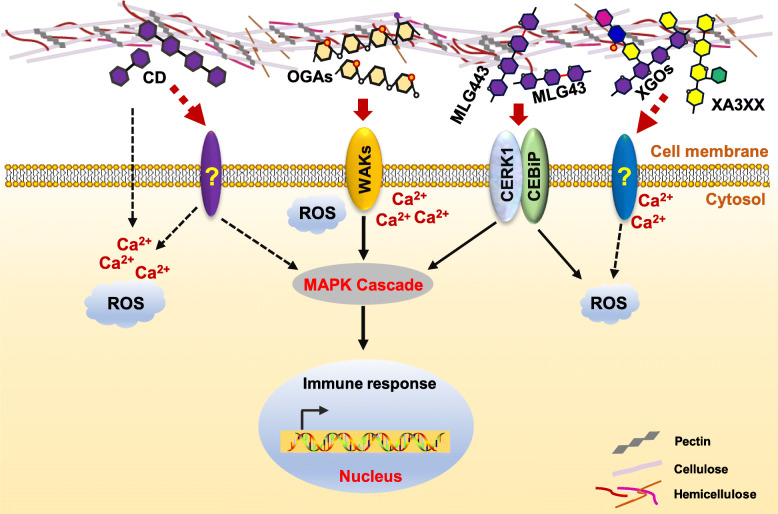
Signaling pathways triggered by cell wall-derived oligosaccharides. Cell wall-derived oligosaccharides function as elicitors recognized by pattern recognition receptor (PRRs) including WAKs, CERK1 and CEBiP to trigger immune response. Cellodextrins derived from cellulose induce the elevation of free cytosolic calcium, generation of ROS and upregulation of *PR* genes. Oligogalacturonic acids derived from pectin are perceived by WAK1 to activate MAPK cascade, which in turn elevates free cytosolic calcium and promotes ROS generation. In rice, trisaccharide 3^1^-β-D-cellobiosyl-glucose (MLG43) and tetrasaccharide 3^1^-β-D-cellotriosyl-glucose (MLG443) are perceived by OsCERK1-OsCEBiP complex, leading to ROS burst, MAPK activation and the expression of immune-responsive genes. Xyloglucan oligosaccharides are able to activate the MAPK cascade, deposition of callose, expression of *PR* genes and increase the biosynthesis of hormones. XA3XX from arabinoinxylan is able to trigger Ca^2+^ influxes, ROS burst, MAPK phosphorylation and the expression of PTI related genes

To gain access into plant cytoplasm and facilitate infection, pathogens need to degrade the plant cell wall, which requires production of a series of CWDEs including cellulase, pectinase, xylanase and xyloglucanase (Fig. 1) (Mary Wanjiru et al. 2002; Lev and Horwitz 2003; Gomez-Gomez et al. 2001; Niture et al. 2006). To combat pathogenic infection, plants have evolved to perceive cell wall fragments released by pathogens for activating defense responses (Jones and Dangl 2006). Those cell wall fragments, released in the form of various oligosaccharides, serve as elicitors of plant immunity (Fig. 2).

### The role of CWDEs in pathogenicity

CWDEs are important for pathogens to breach the plant cell wall without penetration structures or at the late stage of invasion for necrotrophic pathogens (Niture et al. 2006). Considering the complexity of the plant cell wall, it is reasonable that plant pathogens have to produce a set of carbohydrate-active enzymes specifically to degrade plant polysaccharides. At the early stage of invasion, pathogens including *B. cinerea*, *Aspergillus flavus* and *Colletotrichum gloeosporioides* produce polygalacturonase and pectate lyase to depolymerize homogalacturonan of pectin for exposing polysaccharides (D'Ovidio et al. 2004; Lombard et al. 2010; Blanco-Ulate et al. 2014; Kubicek et al. 2014; Yakoby et al. 2000). Other cell-wall degrading enzymes including cellulase, xylanase and xyloglucanase are also able to promote pathogenic infection by degrading cellulose and hemicellulose (van den Brink and de Vries 2011).

Oligosaccharides released from plant cell wall by degradation of polysaccharides can elicit plant defense responses. For example, two endoglucanases MoCel12A/B, belonging to glycosyl hydrolases family GH12, are secreted by *M. oryzae* during infection and are able to degrade rice cell wall mixed-linked glucans. This degradation then releases trisaccharide 3^1^-β-D-cellobiosyl-glucose (G4G3G) and tetrasaccharide 3^1^-β-D-cellotriosyl-glucose (G4G4G3G). G4G3G and G4G4G3G are subsequently perceived by the rice OsCERK1-OsCEBiP complex to promote ROS burst, MAPK activation and the expression of immune-responsive genes for enhancing disease resistance (Yang et al. 2021). Accordingly, *M. oryzae* mutant disrupted with MoCel12A/B are increased in pathogenicity as a result of reduced host immune response (Yang et al. 2021).

In addition to degrading the cell wall, CWDEs also function as effectors to either repress or elicit plant immunity. During infection of rice host, the blast fungus *M. oryzae* secretes a glycosyl hydrolase family 61 protein MoCDIP4 to target the OsDjA9-OsDRP1E protein complex for damping rice immunity. Loss of MoCDIP4 increases fungal pathogenicity due to reduced ability to repress rice immunity (Xu et al. 2020). Five polygalacturonases including BcPG2, BcPG3, BcPG4 and BcPG6 from *B. cinerea*, as well as AnPGB from *A. niger*, are recognized by a leucine-rich repeat receptor-like protein RLP42 in Arabidopsis (Zhang et al. 2014). A xyloglucan-specific endoglucanase PsXEG1 is secreted by *Phytophthora sojae* into the plant apoplast and is recognized by a LRR receptor-like protein response to XEG1 (RXEG1) in *Nicotiana benthamiana* to activate defense response (Wang et al. 2018; Ma et al. 2017). However, the elicitor activity of PsXEG1 does not depend on its xyloglucanase activity (Ma et al. 2015).

### Cellulose-derived oligosaccharide cellodextrins

Cellodextrins are oligomers derived from cellulose and composed of β-1,4 glucoside residues. According to their degree of oligomerization, cellodextrins are present in the form of cellobiose, cellotriose, cellotetraose etc. The polymerization degree of cellodextrins is closely related to their biological activity in eliciting immune response. Cellodextrins are capable of inducing defense responses in grapevine (*Vitis vinifera *L.) including oxidative burst, elevation in free cytosolic calcium and the expression of *PR* genes, thereby enhancing grapevine resistance against *B. cinerea* (Aziz et al. 2007). The cellotriose derived from the endophytic fungus *Piriformospora indica* induces the elevation of calcium, production of reactive oxygen species, changes in membrane potential, and the expression of defense-related genes including *RBOHD*, *MPK3*, *NPR1* and *LOX1 (*Johnson et al. 2018*)*. Pretreatment of Arabidopsis with cellobiose increases resistance to *P. syringae* due to the induction of immune responses including elevation of intracellular calcium, activation of MAPK cascades, but not the generation of ROS or deposition of callose *(*Souza et al. 2017*)*. Upon treatment with cellobiose, cellotriose, or cellotetraose, the typical biotic response gene *WRKY30* is expressed at a similar level in Arabidopsis (Souza et al. 2017). Recently, cellodextrins are reported to be oxidized by an Arabidopsis berberine bridge enzyme-like (BBE-like) protein-CELLOX (cellodextrin oxidase). Transgenic plants overexpressing CELLOX are increased in resistance against *B. cinerea* because the oxidized cellodextrins are less utilized carbon sources for *B. cinerea* (Locci et al. 2019)*.*

### Hemicellulose-derived oligosaccharides

Xyloglucan has also been characterized as potent elicitor of plant immunity. Xyloglucan-derived oligosaccharides are able to activate disease resistance against pathogens and trigger a wide range of defense responses. With respect to tobacco, exogenous addition of xyloglucan oligosaccharides accelerates cell elongation and division, as well as expression of defense responsive genes including jasmonate ZIM-domain gene *JAZ8* and chitinase-like gene *ATHCHIB* (Gonzalez-Perez et al. 2014). In addition, exogenous addition of low concentrations of xyloglucan oligosaccharides changes plant’s response to abiotic stresses, especially cold damage and flooding stress in vine (Salvador and Lasserre 2010). In Arabidopsis, application of xyloglucan increases plant resistance against *B. cinerea* through inducing the expression of various genes involved in defense response, such as MAPK, callose synthase gene *PMR4* and *PR* genes (Claverie et al. 2018).

In Arabidopsis, the pentasaccharide 3^3^-α-L-arabinofuranosyl-xylotetraose (XA3XX) from arabinoxylan is able to trigger immune responses including calcium influx, ROS production, MAPK phosphorylation and expression of PTI-related genes (Melida et al. 2020). Pretreatment with XA3XX confers tomato and pepper with resistance to *P. syringae* and *Sclerotinia sclerotiorum (*Melida et al. 2020*)* Mixed-linked glucans, as another main component of poaceae hemicellulose and some microbial cell wall, can promote defense response as XA3XX. For example, pretreatment of D-cellobiosyl-(1, 3)-β-D-glucose (MLG43) enhances resistance to oomycete *H. arabidopsidis* in Arabidopsis (Rebaque et al. 2021). Similarly, pretreating tomato and pepper with MLG43 before incubation with *P. syringae* and *B. cinerea* respectively confers enhanced resistance (Rebaque et al. 2021)

### Pectin-derived oligosaccharides

The well-studied elicitors derived from plant cell wall are oligogalacturonic acids with different degrees of polymerization produced by degradation of pectin. Oligogalacturonic acids stimulate disease resistance in both grape and Arabidopsis by inducing immune responses including calcium ion flow, accumulation of reactive oxygen species, expression of *PR* genes and changes in protein profiles in apoplast (Aziz et al. 2004; Casasoli et al. 2008; Denoux et al. 2008; Galletti et al. 2008; Ferrari et al. 2007). Consistent with these findings, Arabidopsis and tobacco overexpressing fungal polygalacturonase are increased in resistance to pathogens such as *B. cinerea*, largely due to polygalacturonase-dependent degradation of pectin and release of oligosaccharide elicitors for activating defense response (Ferrari et al. 2007; Ferrari et al. 2008). In addition to pectin, Arabidopsis expressing a fungal cutinase from *Fusarium, solani* f. sp., *pisi* exhibits an alteration in cuticle structure and properties and is changed in postgenital organ fusions (Sieber et al. 2000). Further evidence shows that perturbations in the cutincular layer of transgenic Arabidopsis plants confer full resistance against *B. cinerea* (Sieber et al. 2000; Chassot et al. 2007). This fact could be attributed to the putative products of cuticle upon the reaction of cutinase in triggering defense responses (Chassot et al. 2008).

The plasma membrane associated protein WAK1 has been characterized as the receptor for recognizing oligogalacturonic acids. Its extracellular domain preferentially binds to the de-esterified pectin (Kohorn and Kohorn 2012). Arabidopsis encodes 25 members of the WAK family, among which WAK1 and WAK2 contribute to disease resistance against various pathogens including powdery mildew fungus (Brutus et al. 2010; Kohorn et al. 2009). In rice, overexpression of OsWAK1 improves blast disease resistance (Li et al. 2009), and OsWAK91 interacts with OsWAK92 and OsWAK14 to form dimers for regulating disease resistance (Delteil et al. 2016). There are evidences showing the binding capacity of WAK1 with oligogalacturonic acids (Decreux and Messiaen 2005; Decreux et al. 2006), WAK2 may play role in perceiving oligogalacturonic acids, but it needs evidence.

WAK1 is activated by oligogalacturonic acids with a higher degree of polymerization (DP10-15). Short oligogalacturonic acids (DP4-6, DP1-7) can also induce the expression of *PR* genes in potatoes and tomatoes, despite less effective in activating hormones than long oligogalacturonic acids (Denoux et al. 2008; Ferrari et al. 2007; Davis et al. 1986; Simpson et al. 1998). Very short trimer oligogalacturonic acid DP3 stimulates disease resistance in Arabidopsis at a comparable level as long oligogalacturonic acids. But DP3 results in plant growth retardation (Davidsson et al. 2017). DP3 can promote phosphorylation of MPK3 and MPK6 to trigger immune response without inducing ROS generation (Galletti et al. 2008; Davidsson et al. 2017; Galletti et al. 2011). Partially acetylated oligogalacturonic acids are regarded as more efficient elicitor for activating stress response including the accumulation of phenolic compounds to decrease haustoria formation at the penetration site of *B. graminis* (Randoux et al. 2010). The presence of oligogalacturonic acids with lower degree of methylesterification enhances the resistance to *B. cinerea (*Osorio et al. 2008*)*.

Oligogalacturonic acids generated during the pathogens infection are increasingly recognized to contribute to plant defense response. The global composition of oligogalacturonic acids has been disclosed in Arabidopsis leaves upon infection with *B. cinerea* (Voxeur et al. 2019). The polymerization degree of oligogalacturonic acids produced by fungal pectin lyases ranges from DP3-10, of which mainly are DP4 and DP5. By contrast, the oligogalacturonic acids derived from polygalacturonase are mainly DP2 and DP3, with the DP3 being all methylesterified. Most of the nonmethylesterified oligogalacturonic acids resulted from polygalacturonase are further oxidized probably by OGOX1, a member of Berberine Bridge Enzyme-like (BBE-like) protein family (Voxeur et al. 2019; Benedetti et al. 2018). Oligogalacturonic acids not only participate in immune response, but also regulate the growth and development of plants, because they can inhibit root formation mediated by auxin in Arabidopsis (Savatin et al. 2014*)*. However, it remains largely unknown how oligogalacturonic acids-dependent signaling balances growth and disease response in plants.

### Cell wall-derived elicitors’ recognition by PRRs

Pattern-triggered immunity is initiated when PRRs resident on the plant plasma membrane recognize damage-associated molecular pattern (DAMP) or microbe-associated molecular pattern (MAMP). The oligomers of homogalacturonan, cellulose, mix-linked glucans and arabinoxylan and xyloglucan have been reported as DAMPs in plant immunity (Souza et al. 2017; Claverie et al. 2018; Melida et al. 2020; Rebaque et al. 2021; Aziz et al. 2004). Oligogalacturonic acids are the best characterized DAMP. Lysin motif (LYM) PRR, wall-associated kinase (WAK) and lectin-like (Lec) PRR (mainly for bacterial lipopolysaccharides) are the three main families of receptors involved in the perception of carbohydrate-based DAMPs or MAMPs (Bacete et al. 2018).

The oligogalacturonic acids are percevied by WAK, which contains an extracellular domain called epidermal growth factor (EGF) motif, a transmembrane domain and an intracellular Ser/Thr kinase domain. The N-terminal part of extracellular domain but not the EGF motif confers pectin binding capacity (Brutus et al. 2010) Only WAK1 has been experimentally demonstrated as the oligogalacturonic acids receptor *in vivo* at present (Kohorn and Kohorn 2012). Recently, MLG trisaccharide and tetrasaccharide derived from the rice cell wall have been characterized as novel DAMPs perceived by OsCERK1 and OsCEBiP complex (Yang et al. 2021). MLG trisaccharide is reported to trigger PTI responses partially dependent on CERK1, LYK4 and LYK5 (Rebaque et al. 2021). To date, it remains ambiguous which PRRs detect cellulose-derived oligomers β-1,4-glucans, xyloglucan oligosaccharides and the pentasaccharide of arabinoxylan.

In the course of host-pathogen interaction, the plant can secrete hydrolytic enzymes such as glucanases and chitinases to degrade the cell wall of pathogens. The disrupted fungal cell wall then releases fragments functioning as MAMP to active plant immune responses (Rovenich et al. 2016). Fugal chitin is a well-characterized MAMP (Rovenich et al. 2016). In Arabidopsis, CERK1, together with LYK4 and LYK5, are required for recognizing of chitin oligosaccharides (Liu et al. 2012; Cao et al. 2014). The perception of chitin oligosaccharides in rice depends on OsCEBiP and OsCERK1. OsCEBiP directly binds to chitin oligomers by its LysM domain and forms a receptor complex with OsCERK1 to induce chitin signaling (Liu et al. 2016; Shimizu et al. 2010). As another main component of fungal cell wall, non-branched β-1,3-glucans oligosaccharides from *P. cucumerina* is also a MAMP triggering immune responses of Arabidopsis including the elevation in cytoplasmic calcium, phosphorylation of MAPKs, and upregulation of PTI marker genes (Melida et al. 2018). However, AtCERK1 only functions as a co-receptor, because it does not directly bind to oligosaccharides such as 1,3-β-(Glc)_6_ (Melida et al. 2018; Del Hierro et al. 2021).

DAMPs have been shown to amplify the MAMPs signaling in Arabidopsis. The differentiated outer cell layer of root maintains low expression of PPRs, thus lacking responses to MAMPs. When encountering damage, the epidermal cell upregulates expression of PRRs such as EFR, CERK1, RLP23, FLS2 and LORE in neighbor cells. The upregulation of PRRs subsequently amplifies MAMP responses (Zhou et al. 2020). Intriguingly, DAMPs alone do not induce MAMP responses, suggesting that the DAMPs more likely function in damage perception (Zhou et al. 2020). By integrating the signals of damage and MAMPs, plants are capable of distinguishing non-pathogenic microbes from pathogenic ones (Zhou et al. 2020).

## Conclusion remarks and perspectives

The cell wall varies in both component and structure between different plant species and developmental stages. It undergoes dynamic remodeling throughout plant growth and functions as effective physical barrier to defend against diverse biotic stresses. The cell wall components, including cellulose, hemicellulose, pectin and lignin, are crucial for plant immunity, because mutation of genes associated with either biosynthesis or modification of cell wall leads to changes in disease resistance in many plant species. Besides, cellodextrin, oligogalacturonic acid and xyloglucan oligosaccharide, and those released from cell wall infected by pathogens, have been characterized as potent elicitors or signaling molecules to increase plant disease resistance.

Despite a critical link has been established between the plant cell wall and immunity, there still exist many unknowns regarding molecular regulations of cell wall on immunity throughout plant kingdom. On one hand, it remains ambiguous how plant changes or modifies its cell wall to deal with microbial infection and the underlying mechanisms are largely unknown. On the other hand, it is interesting to determine whether there is a common strategy or specific strategy employed by plants to remodel cell wall for tackling attacks of different types of pathogens. Furthermore, our knowledge on cell wall associated immunity are mostly obtained by studying the model plant Arabidopsis. Less is known about several crops such as rice, wheat and maize.

Increasing number of sequenced crop genomes are available now. The state-of-art biophysical, biochemical and genetic techniques such as CRISPR have been developed. These technical advancements have offered great opportunity to decipher the regulatory role of cell wall modification and composition on plant immunity from a comprehensive and in-depth perspective. Nowadays, it is becoming easier to identify the genes involved in cell wall biosynthesis and modification, allowing us to dissect their regulatory mechanisms on plant disease resistance. Our efforts on studying plant cell wall on immunity will provide not only gene resources, but also fundamental knowledge to develop novel strategies for practically breeding crop cultivars with enhanced disease resistance.

## Supplementary Information

**Additional file 1: Table 1.** Cell wall alteation and corresponding phenotypes against disease.

## Data Availability

Not applicable
